# X-ray induced electrostatic graphene doping via defect charging in gate dielectric

**DOI:** 10.1038/s41598-017-00673-z

**Published:** 2017-04-03

**Authors:** Pavel Procházka, David Mareček, Zuzana Lišková, Jan Čechal, Tomáš Šikola

**Affiliations:** 1 CEITEC - Central European Institute of Technology, Brno University of Technology, Purkyňova 123, 612 00 Brno, Czech Republic; 2 Institute of Physical Engineering, Brno University of Technology, Technická 2896/2, 616 69 Brno, Czech Republic

## Abstract

Graphene field effect transistors are becoming an integral part of advanced devices. Hence, the advanced strategies for both characterization and tuning of graphene properties are required. Here we show that the X-ray irradiation at the zero applied gate voltage causes very strong negative doping of graphene, which is explained by X-ray radiation induced charging of defects in the gate dielectric. The induced charge can be neutralized and compensated if the graphene device is irradiated by X-rays at a negative gate voltage. Here the charge neutrality point shifts back to zero voltage. The observed phenomenon has strong implications for interpretation of X-ray based measurements of graphene devices as it renders them to significantly altered state. Our results also form a basis for remote X-ray tuning of graphene transport properties and X-ray sensors comprising the graphene/oxide interface as an active layer.

## Introduction

Graphene has attracted an enormous attention for its unique mechanical, optical, and electronic properties with a wide technological perspective^1–5^. One of the most appealing graphene attributes is the possibility of controlling the type and concentration of charge carriers via application of an electrostatic potential between a grounded graphene layer and a gate electrode, so called gate voltage. The high intrinsic charge carrier mobility in graphene implies a high application promise for use of gated graphene devices – graphene field effect transistors (GFETs) – as high speed electronic devices^6, 7^. Although a direct application of GFETs in electronic circuits is largely hampered by the lack of a bandgap in a single-layer graphene^8^, there are advanced devices that do not require the bandgap for their functionality. In particular, graphene spintronic devices^9^, gas sensors with sensitivity down to the single molecule limit^10, 11^, and photodetectors^12^ show great potential for future applications.

The research and development of these devices is intimately connected with analysis of their structural, chemical and optical properties. In this respect, the characterization tools based on X-ray radiation are invaluable to determine bond specific chemical composition^13^, graphene-adsorbate charge transfer, molecular orientation, and magnetic properties naming only the most prominent^14^. However, the possible effect of ionizing X-ray radiation on the GFET properties should be considered. In this paper we show that the X-ray radiation induces strong changes in graphene transport properties via charging of intrinsic defects in the gate dielectric.

As the semiconductor field effect transistors (FETs) comprise the heart of a modern electronic industry the huge amount of work has been devoted to understanding their properties with respect to their further development. The quality of the gate dielectric has a profound impact on the long term stability of FETs^15^. More particularly, defects within the dielectric layer behave like charge traps, which can be ionized, e.g., by electron or hole injection or X-ray radiation, rendering FET sensitive to ionizing radiation^16^. The effect of charged impurities and adsorbates is even more pronounced in graphene devices^17, 18^. Recently, the photo-induced doping of graphene has been realized by visible or UV radiation exposure of GFETs^19–26^. Here, two distinct groups of GFET devices were introduced: in the first group the charges excited within the photoabsorbing medium (e.g., MoS_2_, Bi_2_Te_3_, nanoparticles, and plasmonic antennas) are transferred to graphene appearing as an increase of the graphene DC conductivity^19–22^. Within the second group the UV/Vis radiation ionize donor-like traps leaving the gate dielectric positively charged. This charge acts as a positive gate: it increases the electron concentration in graphene by capacitive coupling^23–26^. In contrast to direct graphene doping from adsorbed species causing also a decrease of carrier mobility^27^, the major advantage of “remote gating” is its minimal impact on the charge carrier mobility^24, 26, 28^.

Surprisingly, only little attention was paid to reveal the influence of X-ray radiation on graphene in the GFET configuration. In this respect, Copuroglu *et al*. studied the effect of the gate voltage on the shift of the core-level peaks associated with graphene and gate dielectric using X-ray photoelectron spectroscopy^29^. In a separate work, in pursuit for graphene application as an X-ray sensor, Cazalas *et al*. observed the change in graphene source-drain current of GFET upon hard X-ray irradiation (15 keV) of graphene on SiC^30^. While the latter work introduces the GFET as a device sensitive to X-ray irradiation, the basic description and understanding of the X-ray radiation effect on the GFET is still missing. Here we show that X-ray radiation induces the ionization of donor-like defects in the gate dielectric leading to a large increase of the electron concentration in graphene, i.e., to its strong negative electrostatic doping (n-doping). This results in an observed shift of the position of the charge neutrality point (CNP) to the large negative values. The succeeding irradiation and simultaneous application of negative gate voltage induces backward shift of the CNP as a result of the compensation of positively charged defects by photoexcited electron current in the gate dielectric, an effect which was not demonstrated for visible or UV irradiation so far.

## Results

**Figure 1:**
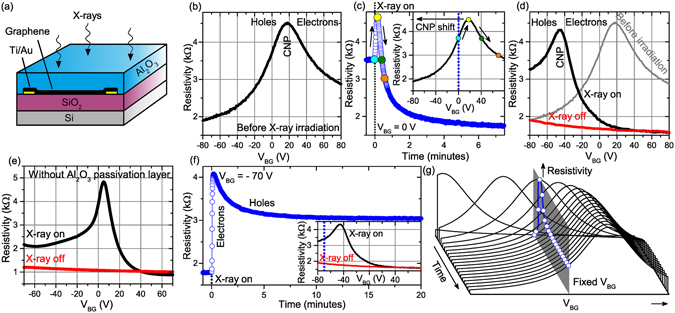
Evolution of GFET resistivity as a function of the back gate voltage V_BG_ (BG trace) or time (time trace), respectively. (**a**) Schematic of the device utilized in this study. All data presented in this figure are obtained for Al_2_O_3_ passivated devices except the case of the panel (**e**). The complementary data for open devices are presented in Supplementary Information, Figure S6. (**b**) BG trace measured for a pristine device (no X-ray irradiation). (**c**) Time trace recorded during the first exposure of the device at V_BG_ = 0 V. The colored circles on the time trace mark the associated position on the BG trace portrayed in the inset. (**d**) BG traces acquired on the pristine (grey) and X-ray irradiated device while the X-ray is on (black) and off (red). (**e**) BG traces acquired for the open (non-passivated) device before (gray), during (black) and after (red) initial X-ray irradiation. (**f**) Time trace measured for V_BG_ = −70 V during succeeding X-ray irradiation. The inset depicts the position of V_BG_ relatively to X-On BG trace. (**g**) Schematic illustration of formation of a time trace via a CNP shift towards more positive V_BG_ values upon succeeding X-ray irradiation. All presented sweeps in the figure are recorded in the direction from negative to positive V_BG_.

The effect of X-ray radiation was studied employing a graphene field effect transistor with a conventional structure consisting of SiO_2_ gate dielectric and Si substrate beneath as schematically shown in Fig. 1a. We have fabricated GFET using a graphene layer prepared by chemical vapor deposition (CVD) on an ultrasmooth copper substrate^31^. Subsequently, the graphene was transferred on a p-doped silicon substrate with a 285 nm thick SiO_2_ layer and pre-fabricated Au/Ti electrodes. Two kinds of devices were used in this study – open and passivated ones. The passivated device comprised a 25 nm thick Al_2_O_3_ passivation layer covering the whole device surface whereas the open are left without the passivation layer. The completed GFET was inserted in an ultrahigh vacuum (UHV) chamber. The exposure to X-rays was carried out by Al Kα radiation generated by a standard X-ray source used for X-ray photoelectron spectroscopy. Both types of the devices showed the qualitatively same type of behavior unless explicitly specified.

The resistivity of passivated graphene as a function of applied back-gate voltage (hereinafter referred to as ‘BG trace’, i.e., back-gate trace) of the GFET device measured before X-ray irradiation is shown in Fig. 1b (see Methods for details). The maximum resistivity, defining the CNP position, is achieved for the back gate voltage V_BG_ = 17 V. Hence, at the zero external bias the graphene layer is positively doped with holes being the majority charge carriers. For V_BG_ larger than 17 V, electrons become the majority charge carriers. In the following we first describe an *initial X-ray irradiation*, which is fundamentally different from the subsequent ones and then we turn our attention to these “*succeeding irradiations*”.

When the GFET is exposed to X-ray radiation at zero back-gate voltage we observe dramatic changes in graphene resistivity in time. As documented in Fig. 1c, the resistivity first increases, reaching its maximum value in 11 s, and then continuously decreases with further irradiation. The maximum value of the resistivity is very similar to the maximum resistivity on the BG trace measured before irradiation, which suggests that the observed change in resistivity is caused by the CNP shift to negative values of V_BG_ as depicted in the inset of Fig. 1c. Indeed, on a separate sample where the X-ray irradiation was sequentially interrupted for measurement after a given X-ray dose we have observed gradual shifts of the CNP towards negative values (see Figure S1 in Supplementary Information). The BG trace measured after a prolonged X-ray irradiation (20 minutes) shows the equilibrium CNP position at V_BG_ = −47 V. The subsequent turning of the X-ray source off portrays the CNP shifted to even larger negative values (Fig. 1d,e), thus indicating a strong X-ray induced n-doping of the graphene layer. In this case, the CNP is already outside the experimentally accessible range of V_BG_ for our GFETs. Further measurements when the X-ray source remains switched off do not show any change in the BG trace even after several hours. If the device was taken out and its BG traces measured, the device returned to the pristine state (V_BG_ = +17 V) only after a 24-hour long exposure of the sample to ambient conditions, which is consistent with the UV/Vis irradiation induced doping effect reported to be stable under vacuum at least for several days^24, 26, 28^.

**Figure 2:**
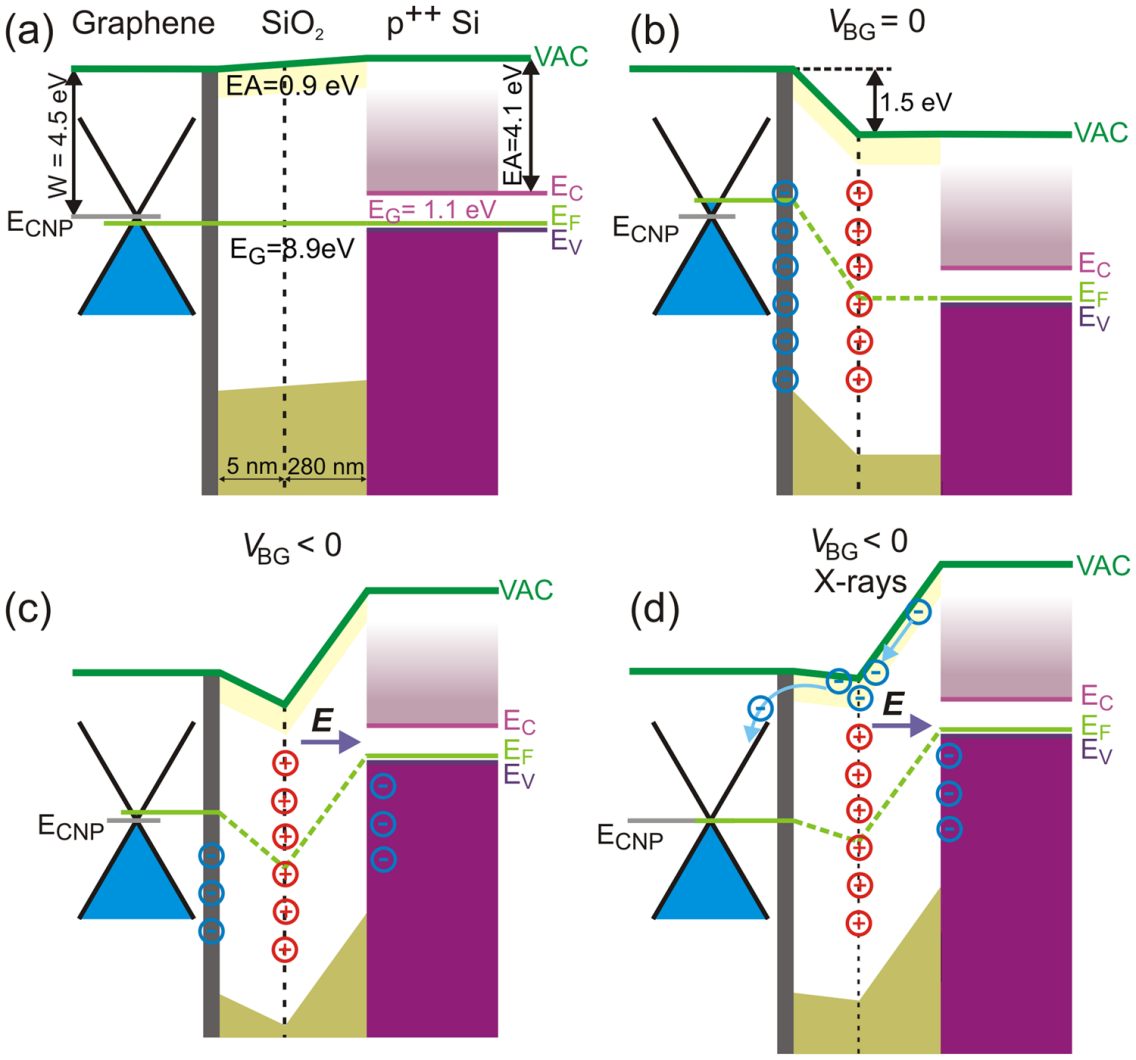
Schematic of the band diagrams of the GFET device. (**a**) The band alignment for the pristine device as adapted from refs 36, 37. (**b**) For the X-ray irradiated device the built in positive charge induces n-doping of graphene and the formation of a potential barrier. (**c**) If the negative V_BG_ is applied to the gate, the potential barrier is partially decreased and transformed into a potential well. (**d**) The photoexcited electrons localized in the SiO_2_ conduction band will be drifted by an applied electric field and accumulate near the location of the positive charge. The accumulated electrons will cause a decrease of the barrier to such an extent so that they can eventually cross it.

Consistently with the recent reports on UV light induced graphene doping^23, 26^, the permanent change of resistivity upon initial X-ray irradiation can be explained by an accumulation of positive charge within the gate oxide, which via capacitance coupling induces negative graphene doping (Fig. 2b). Here, the X-ray radiation is absorbed in the gate oxide and creates initially uniform distribution of the hole-electron pairs throughout the insulator^32, 33^. Depending on the direction of the external electric field, a fraction of electrons and holes recombine and rest of them drift either towards the SiO_2_/Si or graphene/SiO_2_ (G/SiO_2_ in the following) interface. Since electrons possess significantly higher mobility in SiO_2_ than holes they reach either the graphene or the gate on the picosecond time scale whereas the holes diffuse relatively slowly through the oxide layer (micro- to millisecond time scale). Importantly, the SiO_2_ gate dielectric possesses a significant number of donor-like defects ${N}_{D}^{0}$ located primarily 4–8 nm below the G/SiO_2_ interface^23, 33^. Donor-like defects are neutral when they are occupied with electron and can be positively charged when one electron is released (or, equivalently, hole captured). These defect sites can be directly photoexcited to create charged defects (${N}_{D}^{0}\to {N}_{D}^{+}+{{\rm{e}}}^{-}$)^23, 25, 26, 30^ or ionized by capturing diffusing holes (${N}_{D}^{0}+{{\rm{h}}}^{+}\to {N}_{D}^{+}$)^32^. This results in the build-up of the net positive charge ${N}_{D}^{+}$ bound in the gate oxide. This charge is not neutralized during the irradiation due the fact that positively charged defects possess a small cross section for electron capture^33^. The accumulated positive charge can persist for a long time due to the existence potential barrier preventing electrons enter the oxide to neutralize the charged defects^23, 26^. The concentration of charged defect can be determined from the position of CNP, which is, however, outside the observable range. If we assume that the charge carrier mobility in graphene does not change with doping level (which is valid for remote doping^24, 26, 28^) we can extrapolate the CNP position to ~−130 V. From this value we can determine the concentration of charge carries in graphene; this is equal to the concentration of charged defects ${N}_{D}^{+}$ = 1 × 10^13^ cm^−2^ taking into account the direct capacitance coupling of trapped charge in oxide with graphene charge carriers.

By applying an external electric field during the initial X-ray irradiation (on a new device), the system can be driven out of the equilibrium between ${N}_{D}^{+}$ ionization and their neutralization via electron capture (${N}_{D}^{+}+{{\rm{e}}}^{-}\to {N}_{D}^{0}$). At positive V_BG_ electrons are driven out of the near G/SiO_2_ interface region. Hence, in region depleted from electrons there is a lower recombination rate of diffusing holes and, consequently, a higher number of holes approaches this region. The hole diffusion to this region is further promoted by direction of applied external electric field^33^. Both these effects will result in higher hole capture rate and a faster build-up of the positive charge within the oxide and its higher saturated concentration ${N}_{D}^{+}$. In contrast, for V_BG_ < 0, the electric field drives the electrons towards the G/SiO_2_ interface while the holes move in an opposite direction resulting in the exactly opposite effect. These effects are documented by observed increase (decrease) of the n-doping rate and saturated graphene doping level if a positive (negative) gate bias is applied (see Supplementary Information, Section S2). The significant difference in ${N}_{D}^{+}$ charging rate observed for both polarities V_BG_ suggests that ${N}_{D}^{+}$ ionization proceeds rather via trapping of diffusing holes than a direct photoexcitation.

In the following we will describe the succeeding X-ray irradiations; these are fundamentally different from the initial one as there is already a significant population of charged defects in the dielectric layer. During the succeeding X-ray irradiation at V_BG_ = −70 V, the GFET resistivity rapidly increases (Fig. 1f), in 12 seconds its maximum value is reached, and then it slowly decreases. Similarly, to the initial exposure, the changes in resistivity can be explained by the CNP shift towards positive CNP values – in an opposite direction then it has been observed during the initial irradiation; this is schematically represented in Fig. 1g. The BG trace measured during the succeeding irradiation shows that CNP shifts to V_BG_ = −48 V for the passivated device (Fig. 1d). For the open device the observed shift to V_BG_ ≈ +5 V (Fig. 1e) is even higher than for the passivated device. Hence, during the succeeding irradiation the n-doping of graphene is significantly reduced. Here, the CNP reproducibly shifts between X-ray off (X-Off) and X-ray on (X-On) states as shown in Fig. 1d. The observed time evolution of graphene resistivity (time trace in the following) are qualitatively consistent with those measured in an earlier work using harder X-rays (15 keV)^30^. Plotting of the dependence of graphene resistivity on both time and V_BG_ as described in Supplementary Information (Section S3) reveals that the changes in the CNP position after turning the X-ray source on takes place on the time scale of tens of seconds.

We explain the observed shift of the CNP towards positive values during succeeding X-ray irradiations in terms of a transport of photoexcited electrons in the negatively biased oxide towards the G/SiO_2_ interface. These electrons either neutralize or compensate the ${N}_{D}^{+}$ charge. The positively charged donor-like defects ${N}_{D}^{+}$ ionized by X-rays form a large potential barrier preventing electrons to cross the G/SiO_2_ interface as depicted in Fig. 2b. In addition, application of negative V_BG_ creates a potential drop within the gate oxide that leads to (i) a partial decrease of the potential barrier and (ii) its transformation into a potential well (Fig. 2c). Electrons excited by X-rays within the SiO_2_ are drifted by a voltage bias towards the G/SiO_2_ interface and become accumulated in the potential well formed in the vicinity of the G/SiO_2_ interface. The electron accumulation results in a gradual compensation of the positive charge and consequent lowering the energy barrier (Fig. 2d) until electrons are capable to cross it and the electron accumulation reaches the equilibrium level. The decrease of energy barrier to ~0.5 eV would allow the thermal electrons to be transferred to graphene.

The distinct behavior of open and passivated devices originates from the absence/existence of non-gated dielectric near graphene layer, i.e., Al_2_O_3_ layer. In the non-gated Al_2_O_3_ layer there is no electric field and, consequently, no net transport of electrons and the charge in the Al_2_O_3_ is not neutralized/compensated – it behaves exactly as during the initial irradiation with V_BG_ = 0 V. Hence, the neutralization effect in the passivated GFET is only partial (at the SiO_2_ side) and the CNP shifts back only to −48 V instead to 5 V. If we consider that the charge in the SiO_2_ is fully compensated, then the CNP position at −48 V is determined by positive charge localized within the non-gated Al_2_O_3_ passivation layer. The associated defect concentration ${N}_{D}^{+}$ in alumina can be determined from the CNP position to 4 × 10^12^ cm^−2^; ${N}_{D}^{+}$ in SiO_2_ after the initial irradiation therefore amounts to 6 × 10^12^ cm^−2^.

**Figure 3:**
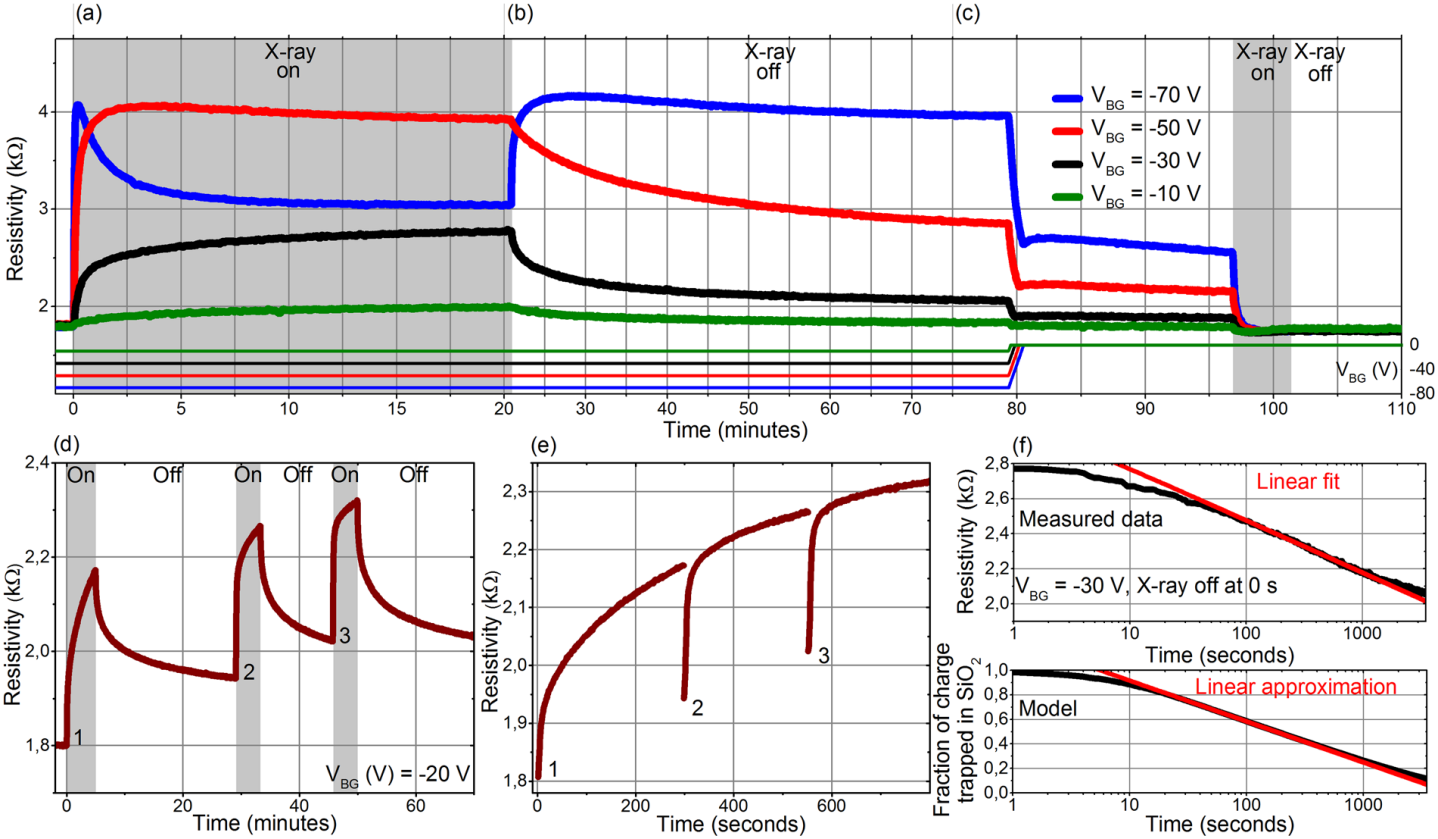
Time traces recorded during succeeding X-ray irradiation and relaxation of the GFETs. (**a–c**) Full time traces showing long (**a**) X-ray irradiation, (**b**) relaxation, and (**c**) erasing. (**d**) Three succeeding X-ray irradiations interrupted with periods of relaxation while keeping the set V_BG_ = −20 V. (**e**) The time trace given in (**d**) with excluded relaxation periods. (**f**) Measured resistivity (top) and calculated trapped charge (bottom) relaxations in logarithmic time scale.

In addition to the charge compensation, the electrons can be directly captured by the positively charged donor-like defects (${N}_{D}^{+}+{{\rm{e}}}^{-}\to {N}_{D}^{0}$) or neutral acceptor-like ones (${N}_{A}^{0}+{{\rm{e}}}^{-}\to {N}_{A}^{-}$). Both these effects will result in the reduction of the trapped charge within the oxide and thus in an associated shift of CNP towards positive voltage values with respect to the X-Off state. Moreover, these effects are rather slow due to a small cross section for electron capture by charged defect states^33^. To reveal the character of the involved defects we have measured the GFET response to the X-ray radiation as a function of time. The time traces measured on passivated devices for negative V_BG_ are shown in Fig. 3a,b. Here, a fast resistivity increase followed by a slow one is also observed. After turning the X-ray source off the device relaxes towards the X-Off state. The resistivity presented in Fig. 3f exhibits a linear dependence in logarithmic scale after certain initial period of time, a behavior typical for tunneling of electrons in SiO_2_ (see Supplementary Information, Section S6)^19, 23, 34^. Moreover, the succeeding X-ray irradiation at both negative and positive voltages renders the device in the different state than was observed with V_BG_ = 0 as shown in Fig. 3a,b. With negative V_BG_ the CNP saturates at −75 V (see also the Supplementary Information, Figure S5). Taking into account the charge in the Al_2_O_3_ passivation layer (4 × 10^12^ cm^−2^) this gives a significant reduction of the ${N}_{D}^{+}$ concentration in SiO_2_ to 2 × 10^12^ cm^−2^. The associated potential barrier for the electron flux to graphene drops to ~0.5 eV, which is comparable to the value enabling electrons to flow to graphene. The positive V_BG_ and simultaneous irradiation causes an additional significant shift of the CNP to larger negative values for both open and passivated devices (see Supplementary Information, Figures S4 and S6d). The rough estimate of CNP shift of ~−50 V in respect to the ~−130 V observed for zero V_BG_ gives the associated ${N}_{D}^{+}$ concentration in SiO_2_ is 9 × 10^12^ cm^−2^ (corrected for a charge in passivation layer). However, one should take this value with care as it was determined from extrapolated data.

The device can be fully returned to the initial state (CNP at ~−130 V) when the V_BG_ is set to zero (Fig. 3c; see Supplementary Information, Figs S3 and S4, for full set of data) and the X-ray source is turned on. The recovery of graphene to the initial state (“erasing”) under certain conditions is observed in all relevant systems featuring radiation induced doping in GFET devices. For G/h-BN stacks and TiO_x_ or MoS_2_ layer the erasing is performed by the long irradiation at V_BG_ = 0^25^ or V_BG_ > 0^19, 24, 26^, respectively. However, in our case the initial state is distinct from the other systems; here, the graphene possesses a strong negative doping observed after an initial X-ray irradiation.

Interestingly, when the succeeding X-ray irradiation was interrupted and after a short period of relaxation renewed, the resistivity quickly increases to the value measured before the turning the X-ray source off and continues as if there was no interruption (Fig. 3d). This is more conveniently demonstrated in Fig. 3e where the relaxation periods were excluded. In contrast, only the initial fast change of the CNP position is observed if the irradiation is carried out with the sample heated to elevated temperatures – after that the CNP position is fixed as results from the constant resistivity in time (see Supplementary Information, Figure S7); the resistivity relaxation after turning the X-rays off is preserved. Based on these observations we infer that the slow increase is associated with an electron capture, i.e., formation of ${N}_{D}^{0}$ or ${N}_{A}^{-}$ and the fast resistivity increase and relaxation is related to the accumulation of thermal electrons in the potential well formed by ${N}_{D}^{+}$ and an external negative bias.

## Discussion

In this paper we have demonstrated that the X-ray irradiation of graphene field effect transistors causes a strong negative doping of the graphene layer as a result of charging of donor-like defects in the gate dielectrics similarly to recently reported graphene doping by visible and UV radiation^23–26^. Since the remote doping does not impose degradation of the charge carrier mobility^24, 26, 28^, this approach possesses great potential in device tuning. We report on the induced doping level on standard SiO_2_ substrates that is much higher than has been observed for the UV doping of G/SiO_2_ or G/h-BN substrates^23, 24, 28^ and comparable both to chemical doping and UV-induced doping employing highly defective TiO_x_ dielectric^26^.

The strong sensitivity to X-ray irradiation has serious implications for long term stability of GFET devices and GFET characterization employing X-ray radiation as a probe. Concerning the later, the advanced strategies should be utilized to obtain the correct results from X-ray based measurements. In this context, a slight negative voltage and continuous sample irradiation will induce the electron accumulation in the near G/SiO_2_ interface volume, thus neutralizing the built-in positive charge rendering the graphene doping level in GFET devices close to intrinsic one.

## Methods

### Graphene preparation

Polycrystalline graphene layers were grown by a standard low-pressure CVD method on ultrasmooth copper foil prepared by the template stripping method^31^. Before the growth, the copper foil was heated up to 1000 °C in a home-built reactor and annealed for 30 minutes in a flow of hydrogen (4 sccm, 10 Pa). The graphene layer was subsequently grown under the methane atmosphere (40 sccm, 70 Pa) for 30 minutes. After cooling the sample to the room temperature, the graphene layer was removed from the bottom side of the copper foil by oxygen/argon plasma treatment (80% O_2_/20% Ar, 0.5 mbar, 2 minutes).

### GFET fabrication

Using a PMMA-assisted wet transfer method^35^, the graphene was transferred to a p-doped silicon substrate (resistivity of 0.00010–0.0015 Ω cm) covered with a 285 nm SiO_2_ thin film (fabricated by dry thermal oxidation by ON Semiconductor) and pre-arranged Au (80 nm)/Ti (3 nm) electrodes fabricated by e-beam lithography. Sputter deposition of metal electrodes before graphene transfer is an important step for fabrication of functional GFET structure. The metal contacts underneath graphene possess rough edges from lift-off process ensuring good graphene adhesion and electrical contact. The prepared structure was inserted into a chip holder and contacted by an ultrasonic wire bonder. The breakdown voltage of the dielectric layer (in full device configuration) in our experiments was higher than 4 MV/cm and leakage current through the oxide at V_BG_ = 80 V was lower than 2 nA. The number of adsorbates attached to the graphene surface was decreased by vacuum annealing of the sample at 150 °C for 1 hour in an atomic layer deposition system (Fiji 200, Cambridge Nanotech), which was subsequently used for deposition of a 25 nm-thick Al_2_O_3_ passivation layer at the same temperature. The Al_2_O_3_ layer was deposited from Trimethylaluminum (TMA, Al(CH_3_)_3_) and H_2_O precursors employing Ar as a carrier gas (pressure in reactor during the growth was 80 Pa and the sample temperature 150 °C. The 25 nm thick layer was grown in 250 deposition/purge cycles each lasting 20 s. The passivation layer enables to exclude the possible side effect of stray electrons produced by an X-ray source or vacuum gauge and adsorption/desorption of impurity molecules to/from graphene surface. The two last technological steps were skipped for the open devices, i.e., those without the passivation layer. The finalized sample was introduced to an ultrahigh vacuum (UHV) chamber equipped with a manipulator providing five contacts to the sample: one used for the gate, two for the source and drain electrodes and the last two for sample heating.

### Transport measurement

Electrical transport measurements were performed under the UHV conditions (base pressure 2 × 10^−7^ Pa) in two terminal geometry, which is sufficient for precise determination of charge neutrality point position. The resistance *R* of the graphene channel between the metal electrodes was measured using a lock-in amplifier SR830 (Stanford Research Systems) with a frequency of 1333 Hz and a fixed current of 100 nA. The graphene resistivity *ρ* was calculated from the channel geometry by the formula *ρ*  = *RW*/*L* where *W* is the channel width (400 μm) and *L* is its length (50 μm). According to the parallel-plate capacitor model, applying the back gate voltage V_BG_ to a silicon substrate results in a change of the charge carrier concentration in graphene by *n* = (*ε*_0_ε_r_/*ed*)V_BG_ where *ε*_0_ is the vacuum permittivity, ε_r_ is the relative permittivity of SiO_2_ (3.9), *e* is elementary charge and *d* is the thickness of the SiO_2_ thin film. Measuring the graphene resistivity as a function of the back gate voltage (BG trace) then gives us an information about graphene doping and charge carrier mobility. Back gate voltage applied to the silicon substrate was changed during the measurements of BG traces with a sweeping rate of 0.7 V s^−1^.

### X-ray irradiation

For X-ray irradiation the non-monochromatic X-ray source (Omicron DAR 400) attached to custom built UHV chamber standardly used for X-ray photoelectron spectroscopy was employed. The photon flux over the sample area was 10^7^–10^8^ photons per second estimated from typical device parameters and sample and source geometry.

## Electronic supplementary material

Supplementary Information
