# Supplementary material for: X-ray induced electrostatic graphene doping via defect charging in gate dielectric
Source: Sci Rep. 2017 Apr 3;7:563. doi: 10.1038/s41598-017-00673-z (PMC5428791; doi:10.1038/s41598-017-00673-z)
Supplement: Supplementary file 1 — Supplementary Information [file 41598_2017_673_MOESM1_ESM.pdf]

SUPPLEMENTARY INFORMATION

X-ray induced electrostatic graphene doping via defect charging in gate dielectric

Pavel Procházka [†‡] *David Mareček,* [‡] *Zuzana Lišková,* [†‡] *Jan Čechal,* [†‡] *and Tomáš Šikola* [†‡]

[†]CEITEC - Central European Institute of Technology, Brno University of Technology, Purkyňova 123, 612 00 Brno, Czech Republic.

[‡]Institute of Physical Engineering, Brno University of Technology, Technická 2896/2, 616 69 Brno, Czech Republic.

[*] E-mail: cechal@fme.vutbr.cz

CONTENTS:

1. Stepwise initial GFET irradiation
2. Initial GFET irradiation at different back gate voltages
3. GFET resistivity under the influence of V_{BG} and X-ray irradiation
4. Open GFET – device without passivation layer
5. Temperature dependence of GFET resistivity during the succeeding X-ray irradiation
6. Charge carrier tunnelling through SiO_2 layer
7. References

1. Stepwise initial GFET Irradiation

To prove that the resistivity change of a GFET device under initial X-ray exposure at V_{BG} = 0 V is caused by a CNP shift, we have used a separate GFET device and measured its BG traces sequentially after particular times of X-ray exposure (t_1–t_4). The shift of the CNP to negative values of V_{BG} is clearly observable in Figure S1a. These results also correspond to resistivity evolution shown in Figure S1b, which was adapted from Figure 1c in the main text.

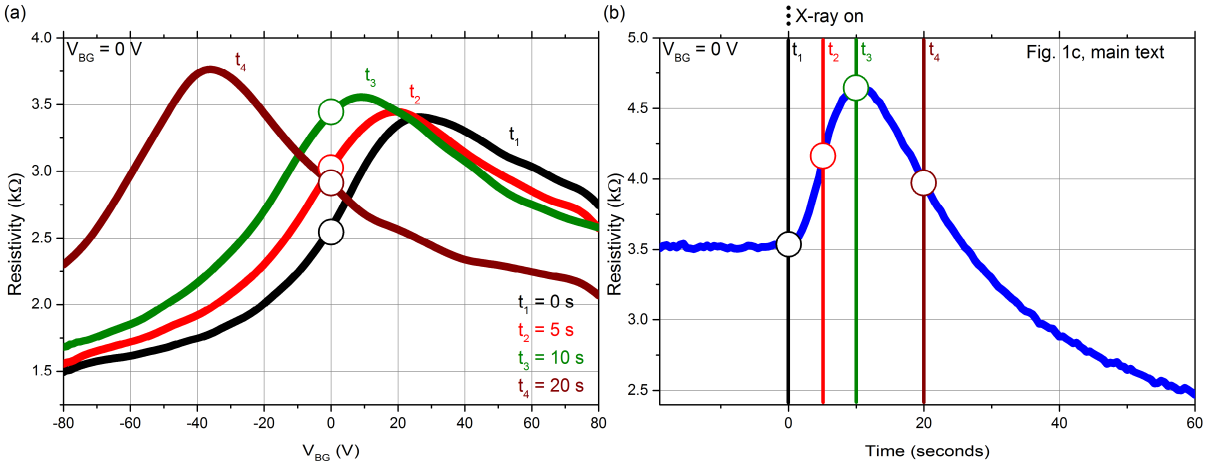

Figure S1: Initial X-ray irradiation of a passivated GFET device. (a) BG traces obtained after particular times of X-ray exposure. (b) Resistivity evolution related to the device from the main text during its initial irradiation at V_{BG} = 0 V. The circles in the graph schematically indicate corresponding resistivity values in the BG traces shown in (a).

2. Initial GFET irradiation at different back gate voltages

Time traces measured for initial X-ray irradiation of an open graphene device are given in Figure S2a. When the negative V_{BG} is applied during the initial X-ray irradiation we observe only a slight change in resistance and an associated shift in the CNP position as evident from Figure S2b. In contrast the applied positive V_{BG} enhances the rate of the CNP shift and strong negative graphene doping is observed after turning the X-ray source off. For the succeeding irradiation and measurement of the BG trace the resistivity maximum is observed near zero V_{BG} (Figure 2d).

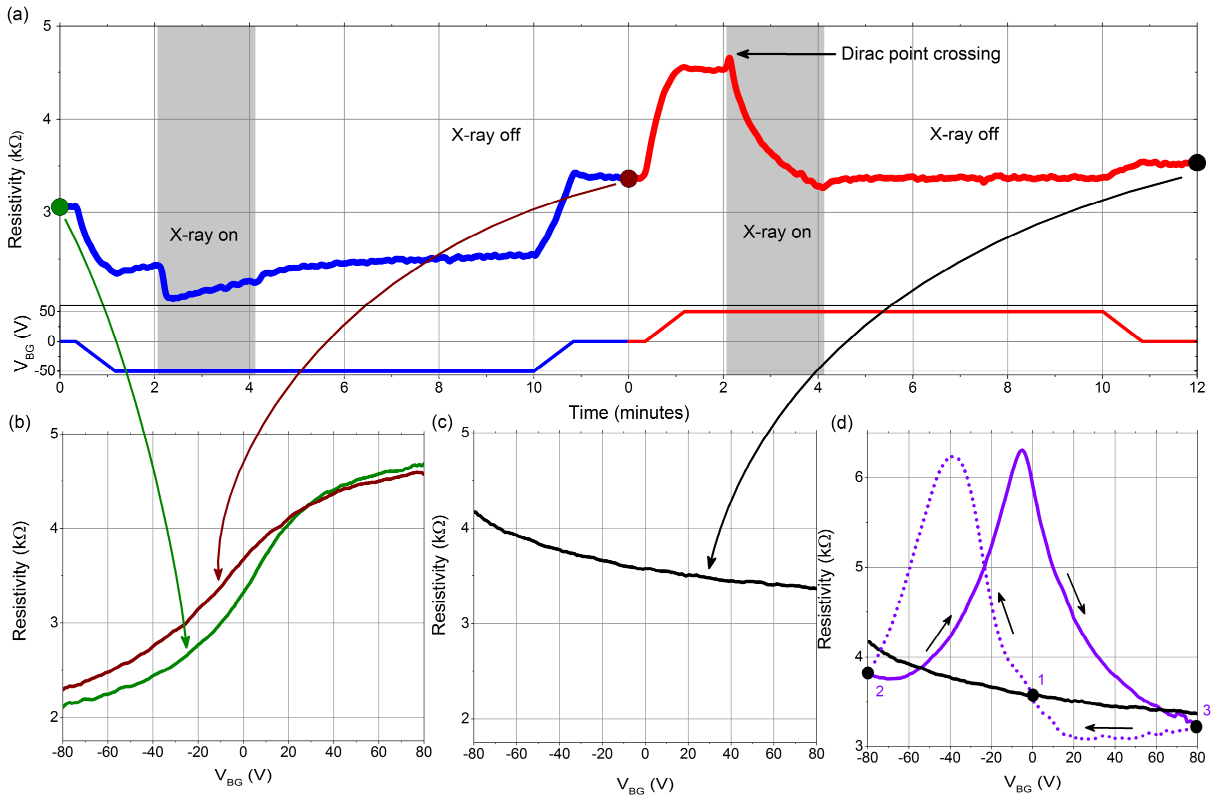

Figure S2: (a) Time trace measured during the initial X-ray exposure of an open device at V_{BG} = -50 V (blue) and subsequently at V_{BG} = 50 V (red). (b, c) BG traces measured before and after irradiation at V_{BG} = -50 V (b) and after irradiation at V_{BG} = 50 V (c). The points on the time trace mark when the BG trace was measured. (d) BG traces measured during succeeding X-ray irradiation carried out immediately after the initial irradiation period. The numbered points mark the sequence of measurements.

3. GFET resistivity under the influence of V_{BG} and X-ray irradiation

Figures S3 and S4 summarize the time traces measured for a passivated device. Each trace contains three regions: X-ray irradiation, relaxation and erasing at $V_{BG} = 0$ V with X-ray source on.

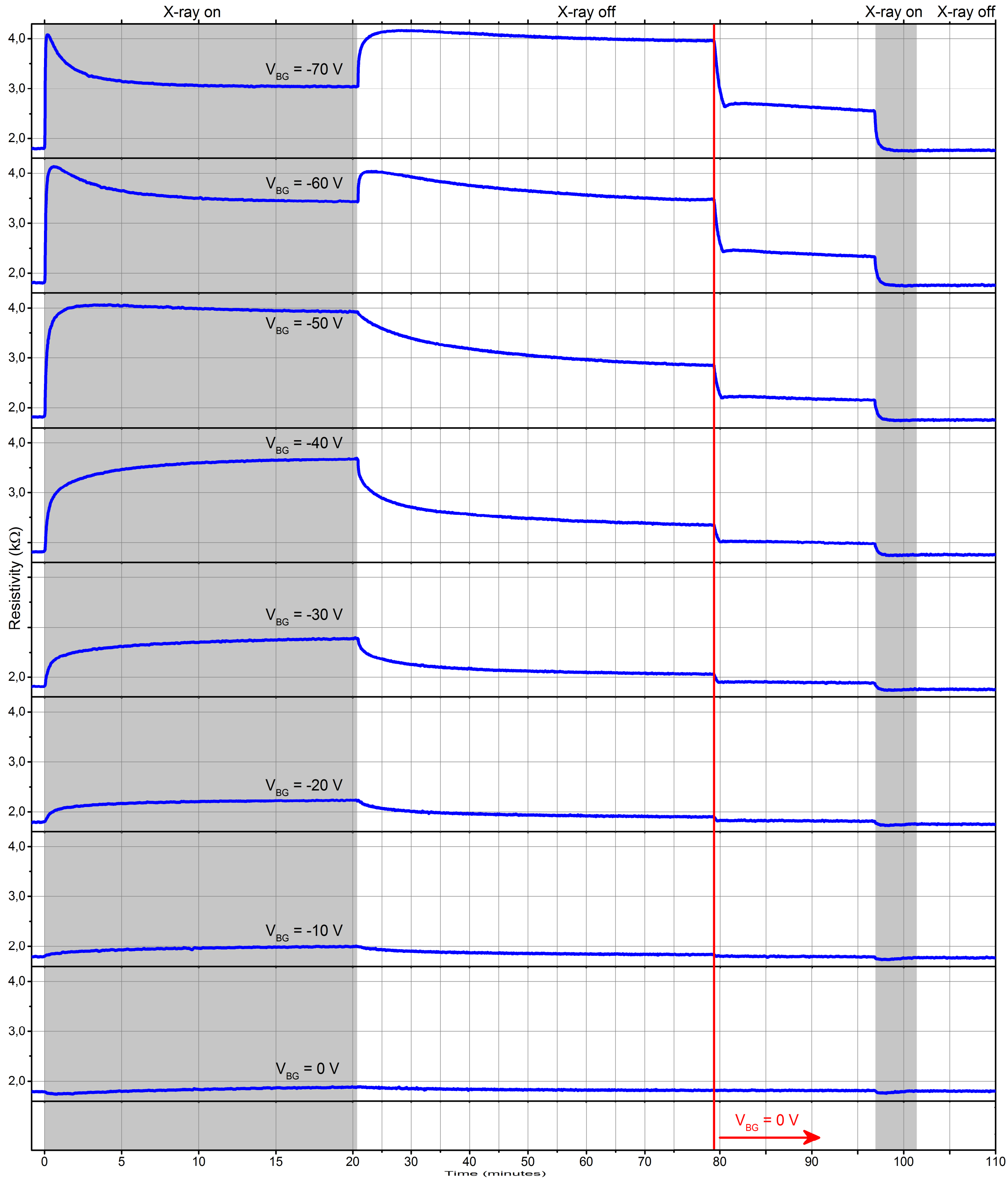

Figure S3: Complete set of time traces measured for a passivated sample and negative V_{BG}.

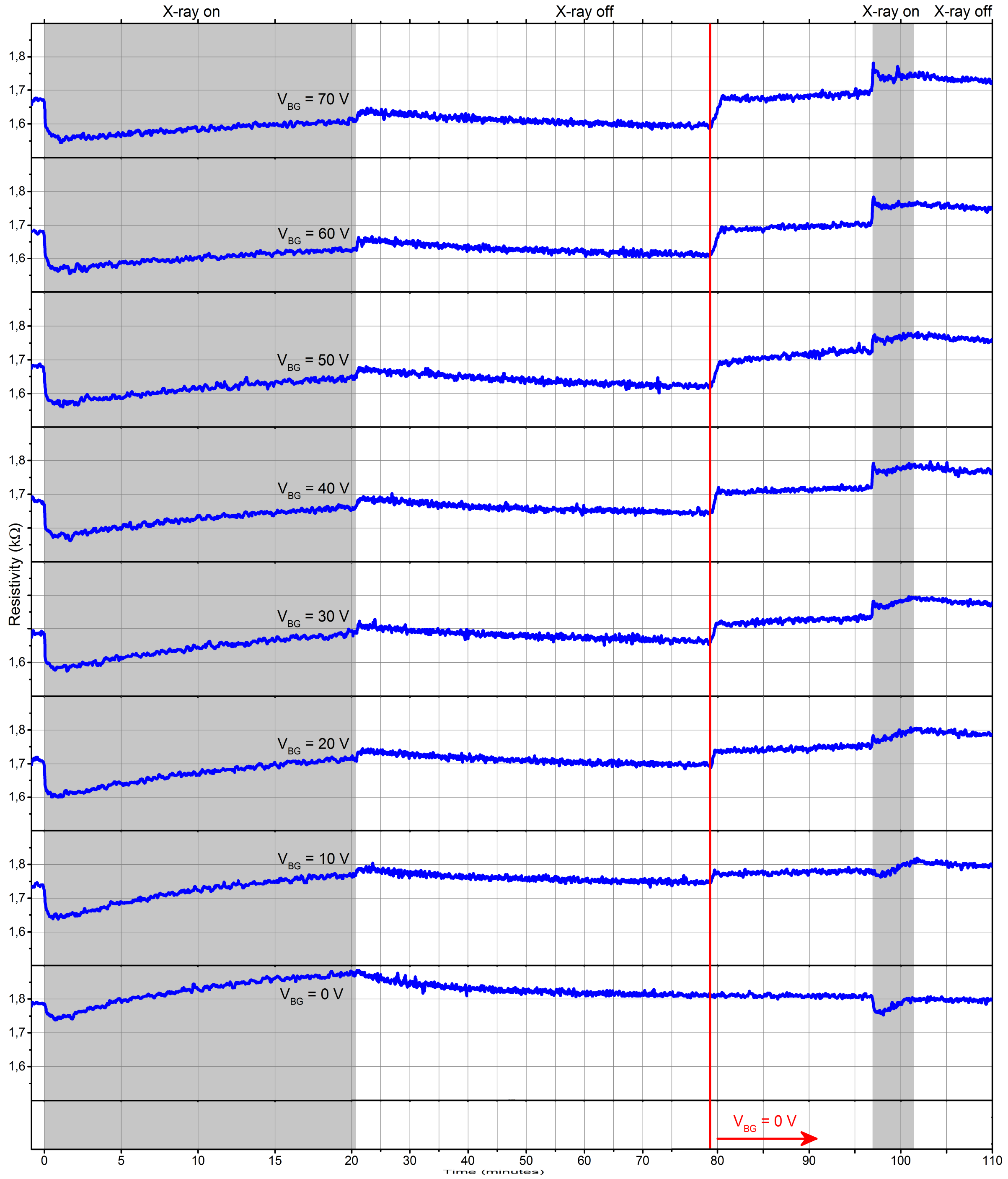

Figure S4: Complete set of time traces measured for a passivated device and positive back gate voltages. Please note the different resistivity scale is used than in Figure S3.

Plotting the set of time traces for back gate voltages in the range from -70 to 70 V as a function of both time and V_{BG} we are able to reconstruct the time evolution of BG traces as depicted in

Figure S5. It is evident that the changes in the CNP position after turning the X-ray source on are very fast. Within the first 30 s the CNP reaches the value of -60 V and then more slowly converges to the equilibrium X-On CNP position at V_{BG} = -47 V. The same value is measured for the X-On BG trace (see Figure 1d in the main text). The initial widening of the resistivity maximum can be ascribed to the different rates of CNP shifts for different values of BG voltage. When the CNP reappears, i.e. for X-ray irradiation times longer than 12 seconds we can calculate the mobility for a specific charge carrier concentration. When the X-ray is turned off after 20.9 minutes the CNP does not shift back to X-Off state (~ -130 V) but to ~ -75 V.

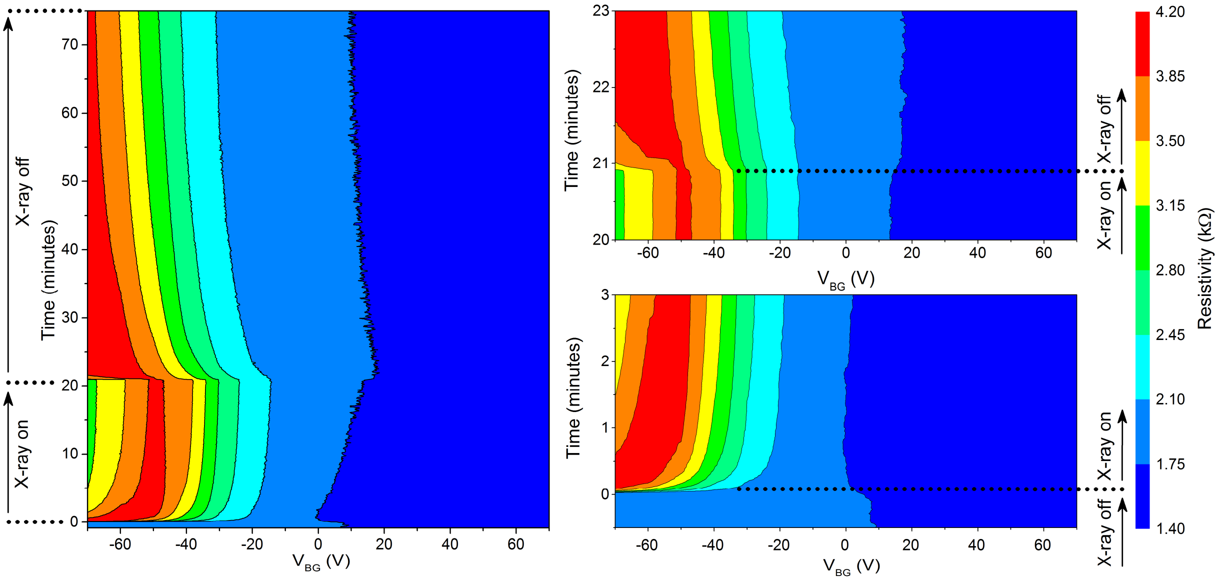

Figure S5: Color-coded graphene resistivity as function of V_{BG} and time reconstructed from the individual time traces depicted in Figure S3 and S4. The panels on the right show a magnified view shortly after turning the X-ray on (bottom) and turning it off (top).

4. Open GFET – device without passivation layer

Data obtained for the open (non-passivated) device are shown in Figure S6.

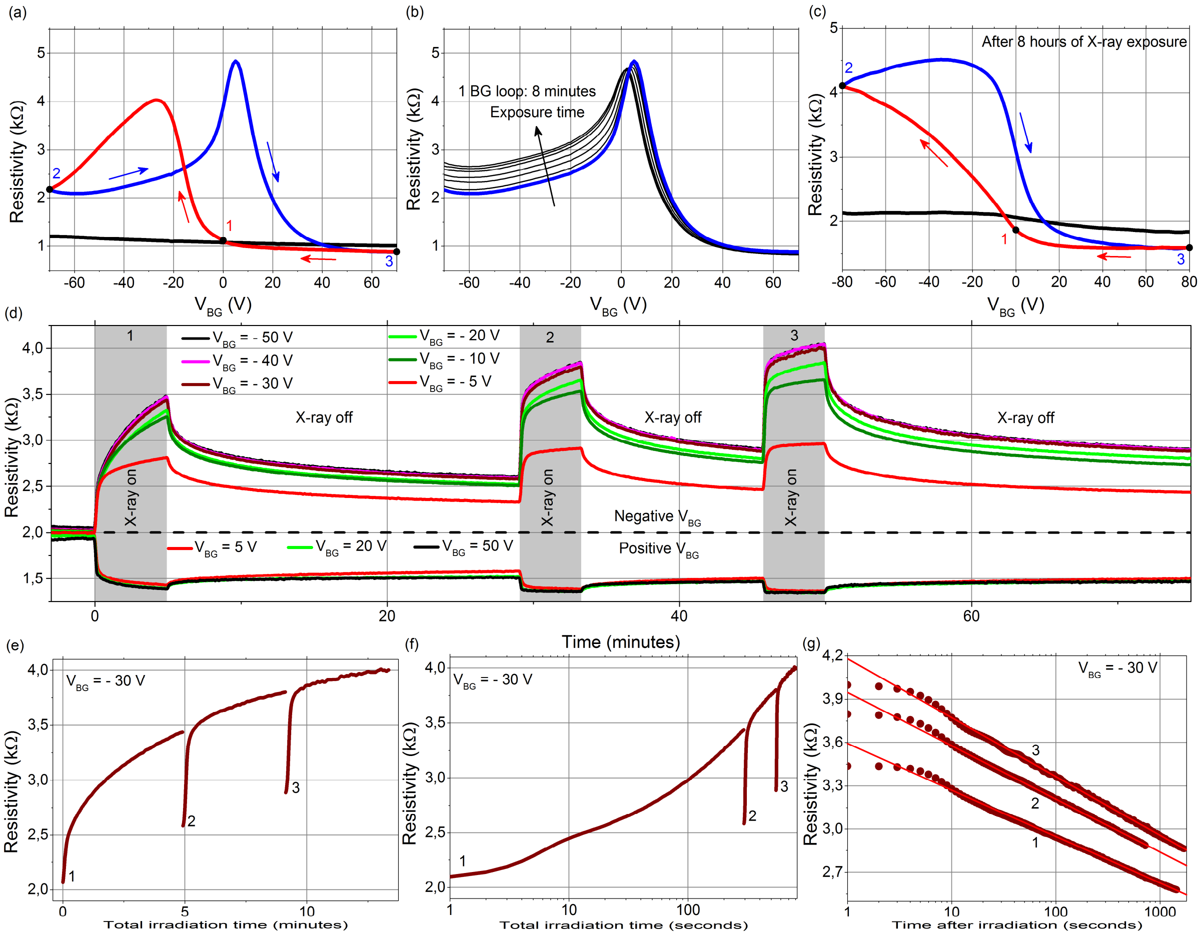

Figure S6: Complementary data as shown in Figs. 1 and 3 (the main text) but for the open device. (a) Resistivity of the GFET structure as a function of V_{BG} (BG trace) measured after the initial X-ray irradiation (black) and during the succeeding irradiation (red and blue). The measured hysteresis is marked by the arrow and the different colors mark the different sweep direction; the measured sequence in marked by numbered points. (b) Evolution of BG trace in time during continuous X-ray irradiation. (c) Final BG trace measured after the prolonged X-ray irradiation. (d) Time traces measured for negative and positive V_{BG}. (e) Increase in the resistivity of the device taken from (d) excluding the relaxation periods. The numbers denote the marked time periods in panel (d). (f) The same figure plotted in a logarithmic time scale. (g) Resistivity decrease during relaxation periods in (d) plotted in logarithmic time scale.

During the prolonged irradiation the continuous degradation of the hole mobility is observed for the open device (Figure S6b) resulting in almost monotonic BG trace (Figure S6c). This can be understood in terms of continuous build-up of negatively charged defect states N_{A}^{-} at the graphene/SiO_2 interface as for negative V_{BG} these trap sites become filled ($N_A^0 + \mathrm{e}^- \rightarrow N_A^-$) and their negative charge causes strong scattering of carriers of the opposite charge, i.e. holes in graphene.[S1] Alter very long irradiation we observe almost a flat BG trace swept from negative values to zero V_{BG}, where these traps become empty and we observe drop in the BG trace. Hence, the BG trace measured with X-ray on does not contain only the information on pristine graphene but also on additional dynamical effects similar to those causing hysteresis in the GFET devices. We note that this behavior is largely suppressed by the presence of an Al_2O_3 overlayer.

5. Temperature dependence of GFET resistivity during the succeeding X-ray irradiation

Time traces measured for the passivated device at room and elevated temperatures are depicted in Fig. S7. The sample heating was provided by a standard BN heater attached to the sample holder.

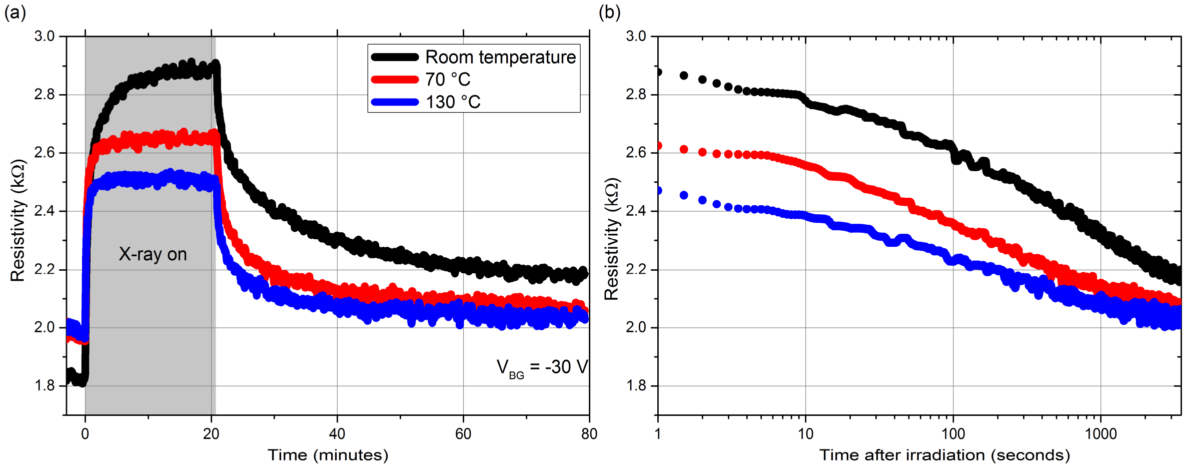

Figure S7: Time traces obtained for succeeding X-ray irradiations at V_{BG} = -30 V and different substrate temperatures.

6. Charge carrier tunneling through a SiO_2 layer

To provide a more quantitative insight to the observed phenomena we have studied the relaxation of resistivity of GFETs after turning off the X-ray source. The GFET resistivity measured at V_{BG} = -30 V as a function of the logarithm of time after turning X-ray off is presented in Figure S9a (and also in Figure 3c in the main text). In semiconductor FETs, this behavior is typical for tunneling of trapped charges from a dielectric substrate to the channel or gate. Adapting the theory devised for these devices we can quantitatively reproduce the measured dependence. Considering the uniform initial distribution of trapped charge and considering the given tunneling probability (between the graphene the gate oxide) we can derive the equation for time evolution of trapped charges in the gate dielectric (see below for detailed derivation):

$$\sigma(t) = \sigma_i \left[1 - \left(\ln \frac{\tau_d}{\tau_0} \right)^{-1} \ln \frac{t}{\tau_0 e^{-\gamma}} \right],$$

where σ_i is the total number of trapped charges in the dielectric substrate, τ_d and τ_0 are the constants related to their tunneling probability through the oxide, and γ = 0.577 is Euler's constant. Using this equation, we can fit the dependence of graphene resistance presented in Figure S9. The logarithmic dependence of the relaxation in the time domain is typical for tunneling of electrons in SiO_2.[S2, S3] This may concern both electrons entering the gate oxide to annihilate positive defects[S4] or electrons detrapped from acceptor like defects tunneling to graphene.

Detailed derivation:

In the model adapted for neutralization of trapped charges we assume their uniform density N in SiO_2 as depicted in Figure S4a. Charge trapped at a distance x from the surface has a time constant to be neutralized by charge on the surface

$$\tau(x) = \tau_0 \exp(\alpha x), \tag{1}$$

where τ_0 is the time constant of the charges trapped on the surface (Figure S8b, x = 0) and α is the constant related to the probability of charge tunneling through the oxide.[S2, S5]

After time t the charge at the distance x decreases to $e^{-t/\tau(x)}$ of its initial value and the total charge in SiO_2 is

$$\sigma(t) = \int_0^d -qNe^{-t/\tau(x)}\,\mathrm{d}x, \tag{2}$$

where d is the thickness over which the neutralization is effective (4 – 8 nm). The initially stored charge in SiO_2 is therefore

$$\sigma_i = \sigma(0) = -qNd. \tag{3}$$

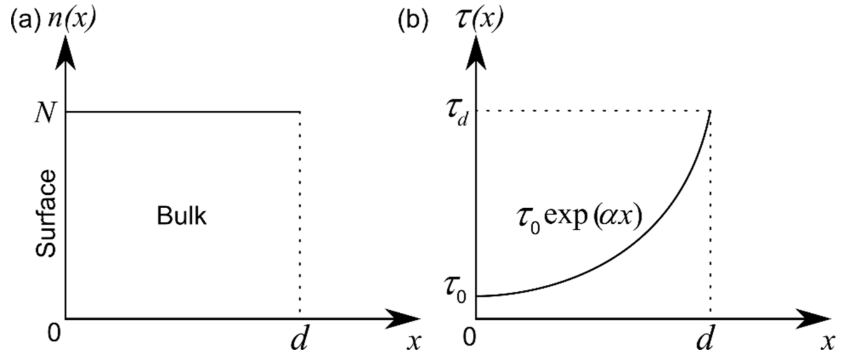

Figure S8: (a) Density of trapped charges n in the SiO_2 layer as a function of the distance x from the surface. (b) Dependence of the characteristic time τ needed for trapped charges at a distance x to be neutralized.

Using equation (1) we get

$$\mathrm{d}\tau = \alpha\tau_0 \exp(\alpha x)\,\mathrm{d}x = \alpha\tau\mathrm{d}x, \tag{4}$$

and hence

$$\sigma(t) = \int_{\tau_0}^{\tau_d} -qNe^{-t/\tau}\frac{1}{\alpha\tau}\mathrm{d}\tau = \frac{\sigma_i}{\alpha d}\int_{\tau_0}^{\tau_d}\frac{e^{-t/\tau}}{\tau}\mathrm{d}\tau, \tag{5}$$

where $\tau_0 = \tau(0)$ and $\tau_d = \tau(d) = \tau_0 e^{\alpha d}$.

Solving equation (5) using the exponential integral Ei we get

$$\sigma(t) = \sigma_i\left(\ln\frac{\tau_d}{\tau_0}\right)^{-1}\left[Ei\left(-\frac{t}{\tau_0}\right) - Ei\left(-\frac{t}{\tau_d}\right)\right], \tag{6}$$

which can be approximated by

$$\sigma(t) = \sigma_i, \qquad (t < \tau_0) \tag{7}$$

$$\sigma(t) = \sigma_i\left[1 - \left(\ln\frac{\tau_d}{\tau_0}\right)^{-1}\ln\frac{t}{\tau_0 e^{-\gamma}}\right], \qquad (\tau_0 < t < \tau_d) \tag{8}$$

where $\gamma = 0.577$ is Euler's constant.

A comparison of the resistivity evolution of GFET device measured after turning the X-rays off at V_{BG} = -30 V with the model is presented in Figure S9. Equation (8) describes the linear part of the time logarithm dependence, while equation (6) gives the deviations from this behavior. We point out that the linear approximation is valid only in the time scale $\tau_0 < t < \tau_d$ and the deviations are related to the time constants τ_0 and τ_d.

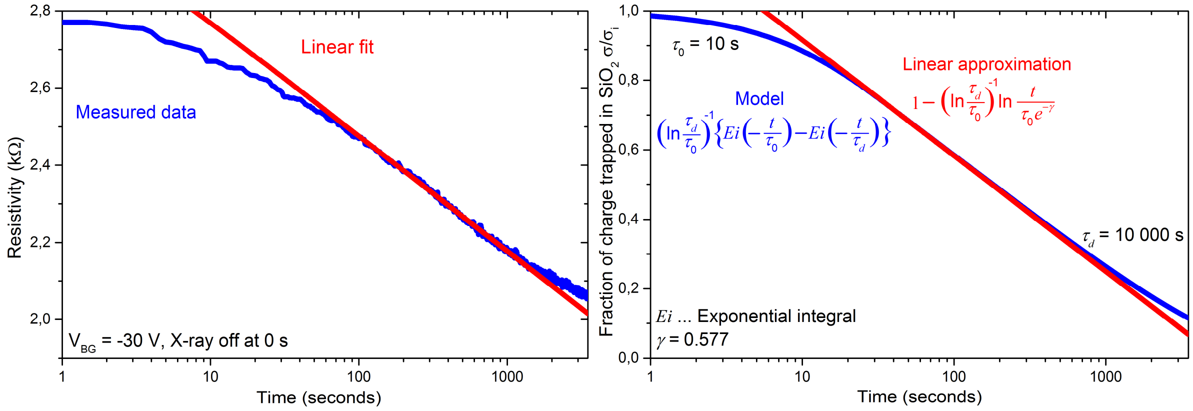

Figure S9: (a) Experimental time dependent GFET resistivity after turning off the X-ray source at V_{BG} = -30 V. The red line was used to fit a linear part of dependence. (b) Corresponding model for neutralization of trapped charges in SiO_2 from equation (6) and its linear approximation from equation (8) calculated for constants τ_0 = 10 s and τ_d = 10 000 s. These times are comparable with those valid for electron tunneling into SiO_2 from Si.[S2]

7. References

S1. Farmer, D. B.; Golizadeh-Mojarad, R.; Perebeinos, V. Lin, Y-M; Tulevski, G. S.; Tsang, J. C., & Avouris, P. *Nano Lett.* **9**, 388–392 (2009).

S2. Lakshmanna, V. & Vengurlekar, A. S. *J. Appl. Phys.* **63,** 4548–4554 (1988).

S3. Roy, K. *et al. Nat. Nanotechnol.* **8,** 826–830 (2013).

S4. Kim, Y. D. *et al. ACS Nano* **7,** 5850–7 (2013).

S5. Yamabe, K. & Miura, Y. *J. Appl. Phys.* **51,** 6258–6264 (1980).
